# Are online search trends associated with changes in dental sealant utilization?: focusing on key influencing factors

**DOI:** 10.1186/s12903-026-08733-5

**Published:** 2026-06-23

**Authors:** Hye Young Mun, Kyuseok Kim, Jung Yun Kang

**Affiliations:** 1 Department of Dental Hygiene, College of Health Sciences, Yonsei University, Wonju, 26493 Republic of Korea; 2 Department of Artificial Intelligence Software, Korea Polytechnics, Seongnam, Republic of Korea; 3 Department of Oral Biology, Yonsei University College of Dentistry, Seoul, Republic of Korea

**Keywords:** Consumer health information, Dental caries prevention, Dental health services, Dental sealants, Information-seeking behavior, Preventive dentistry

## Abstract

**Background:**

Dental sealants are an important preventive service for reducing occlusal caries in children and adolescents. As digital health information–seeking behaviors have become increasingly prevalent, online search activities may serve as indicators of population-level interest in preventive healthcare services. Understanding how online search trends relate to actual dental sealant utilization may provide valuable insights for public health planning.

This study aimed to examine longitudinal trends in dental sealant utilization and online search activity in South Korea from 2016 to 2023 and to assess the association between sealant utilization and online search trends.

**Methods:**

Monthly data on patients receiving dental sealants under the National Health Insurance program were analyzed alongside relative search volume (RSV) data for “dental sealant” obtained from NAVER Data Lab. Subgroup differences in online search activity according to sex, age group, and search device were assessed. Multivariate ordinary least squares regression was performed to examine the association between RSV and sealant utilization, with adjustment for key influencing factors.

**Results:**

Sealant utilization exhibited pronounced seasonal variation, with higher utilization during school oral examination and vacation season. Significant differences in online search activity were observed across sex, age groups, and search devices, with the highest search interest observed among individuals aged 19–39 years. Over time, mobile-based searches steadily increased and surpassed desktop-based searches. RSV was positively and significantly associated with sealant utilization after adjustment for covariates (*P*=.036). Larger population size and higher household income were associated with greater sealant utilization, whereas a higher proportion of individuals with lower educational attainment was associated with reduced utilization.

**Conclusions:**

Online search activity was significantly associated with dental sealant utilization, suggesting that digital search trends may reflect information-seeking behavior at the population level. The increasing predominance of mobile-based searches highlights the importance of mobile-centered digital strategies for disseminating evidence-based oral health information. Integrating online search indicators with traditional utilization data may support the timing, targeting, and design of preventive dental health policies and outreach programs.

**Supplementary Information:**

The online version contains supplementary material available at 10.1186/s12903-026-08733-5.

## Introduction

Dental caries remains one of the most common preventable oral diseases among children and adolescents and continues to represent a major public health burden [1]. Dental sealants are an evidence-based preventive intervention that reduce the risk of occlusal caries by protecting pits and fissures of permanent molars from food debris and cariogenic microorganisms [2–4]. Because newly erupted permanent molars are particularly susceptible to caries, timely sealant application during childhood and adolescence is important for preventing disease progression and reducing future treatment needs [5].

In Korea, dental sealants have been incorporated into the national health security system, primarily through the National Health Insurance, to improve access to preventive dental care for eligible children and adolescents. Since the introduction of partial National Health Insurance coverage for first permanent molars in 2009, coverage was expanded to first and second permanent molars for individuals younger than 18 years in 2013, and patient co-payment was reduced in 2017 [6, 7]. These policy changes reduced financial barriers to sealant use. However, insurance coverage alone may not ensure uniform utilization, as preventive service use can still be influenced by parental awareness [8], regional accessibility and public health infrastructure [7], and exposure to relevant health information [9–11]. Therefore, examining both real-world utilization and public information demand may provide additional insight into population-level preventive dental service use.

Dental sealants are directly relevant to dental public health because they prevent occlusal caries in children and adolescents, a population in which early prevention can reduce future restorative treatment needs and oral health inequalities [2–5, 12]. For children and adolescents, decisions regarding preventive dental care are usually made by parents or guardians [8]. Health literacy and digital literacy may therefore influence whether parents recognize the risk of caries, understand the preventive value of sealants, trust professional recommendations, and seek or accept timely care for their children [13–15]. In this context, online search interest regarding dental sealants may reflect population-level information demand related to caries prevention and preventive service use [9–11, 16, 17].

In Korea, Naver DataLab provides aggregated relative search volume data derived from user search queries on the Naver platform [18–20]. Therefore, the present study used Naver DataLab as an analytical source for assessing temporal changes in public search interest, rather than as a direct measure of individual browsing or information-seeking behavior. In infodemiology, online search data have been used to explore public attention, information demand, perceived risk, and temporal patterns related to health issues and healthcare services [17, 21]. Although search volume data do not directly measure individual health literacy or disease prevalence, they may capture population-level online attention toward specific health services [16, 17]. In the present study, relative search volume was therefore used as an infodemiologic indicator of information demand regarding dental sealants and was examined in relation to real-world sealant utilization.

Previous studies have examined dental sealant utilization, insurance coverage, and oral health inequalities in Korea [9, 12, 22]. However, limited evidence is available on whether population-level online search interest regarding dental sealants corresponds with real-world sealant utilization trends. Addressing this gap may provide useful insight into how public information demand is associated with preventive dental service use and may inform population-level strategies to improve timely sealant utilization. Therefore, this study aimed to analyze temporal trends in dental sealant utilization and Naver relative search volume for dental sealants in Korea from 2016 to 2023 and to assess the association between online search interest and sealant utilization.

## Methods

### Study design

This study was designed as a retrospective longitudinal ecological study using secondary administrative health insurance claims data and online search trend data. The conceptual framework of this study was based on the assumption that online public interest in dental sealants, measured using relative search volume (RSV), may be temporally associated with the utilization of preventive dental services.

Because data on online search interest became available in 2016, the analysis was conducted using data from January 2016 to December 2023, representing an 8-year investigation of dental sealant utilization trends and temporal changes in public interest. The study population consisted of 5,853,477 patients aged 5–18 years who received dental sealant applications under the NHI coverage. Data were analyzed by sex, age, region, and type of medical institution to examine trends in sealant utilization over the study period. To assess variations in online interest, RSV was analyzed according to sex, age group, and search device. The association between the monthly number of patients receiving sealants and RSV was analyzed longitudinally to compare temporal trends.

The monthly number of patients receiving dental sealants was defined as the outcome variable, RSV was defined as the main exposure variable, and demographic, socioeconomic, seasonal, healthcare supply, and epidemiological factors were included as control variables. A multivariate regression model was constructed incorporating population size, number of dental institutions, annual household income, educational attainment, vacation season, school oral examination periods, and the monthly number of confirmed COVID-19 cases as adjustment variables. This approach was used to evaluate the potential influence of RSV on sealant utilization and to clarify the effect of online public interest on the actual use of preventive dental services.

### Data and variables

The monthly number of patients receiving dental sealants was defined as the dependent variable and extracted based on procedure code U2390 from the Health Insurance Review and Assessment Service [6]. These data represented patients aged 5–18 years who received dental sealant applications under NHI coverage during the study period. The data were analyzed at the monthly aggregate level and did not include personally identifiable information.

RSV derived from Naver Data Lab search data was used as the independent variable [18]. Monthly RSV data were collected from January 2016 to December 2023 by setting the time unit to “month” in Naver DataLab. RSV represents a normalized relative search index, in which the highest search volume within the specified search period and keyword group is assigned a value of 100, and the remaining monthly values are expressed relative to that peak. RSV was analyzed according to sex, age group, and search device; in these analyses, relative values were normalized to enable comparison across subgroups. The search terms used to assess RSV are presented in Table S1.

To identify additional factors influencing sealant utilization rates, the following control variables were set based on the literature: demographic, socioeconomic, seasonal, and epidemiological factors. Data were obtained from the Korean Statistical Information Service National Statistics portal [23]. The control variables included the population aged 5–18 years [24], the number of dental institutions [25], annual household income [7, 26], education level [7, 27], vacation season [28], the timing of annual school dental examinations [29], and the COVID-19 pandemic [30]. The number of confirmed COVID-19 cases was included because the COVID-19 pandemic overlapped with the study period and may have affected both healthcare utilization and information-seeking behavior. Major public health campaigns or promotional activities related to dental sealants were not included as separate variables; however, vacation seasons and school oral examination periods were included as seasonal contextual factors that may explain temporal variations in sealant utilization. The RSV search terms, operational definitions, and data sources of all study variables are summarized in Table S1.

All variables were collected on a monthly basis. Variables with different collection cycles were preprocessed and converted into a monthly format to ensure consistency across datasets. Data collection and preprocessing were completed in January 2025. As the study involved secondary analysis of anonymized data, informed consent was waived. The study protocol was reviewed and determined to be exempt from review by the Institutional Review Board of Yonsei University Mirae Campus (approval number: 1041849-202501-SB-015-01). All data were handled at an anonymized and aggregate level, and no personally identifiable information was accessed during data collection, preprocessing, or analysis.

### Statistical analysis

Trend analyses of the number of patients receiving sealants and RSV were conducted using independent samples t-tests and one-way ANOVA, depending on the variable type. To assess the effect of RSV on sealant utilization, multivariate ordinary least squares (OLS) regression analysis was performed. In this model, the monthly number of patients receiving dental sealants was specified as the dependent variable, RSV was specified as the main independent variable, and population size, number of dental institutions, annual household income, educational attainment, vacation season, school oral examination period, and the monthly number of confirmed COVID-19 cases were included as control variables. Statistical significance was defined as α = 0.05. Trend analyses were conducted using GraphPad Prism 8.0 (GraphPad Software, San Diego, CA, USA), whereas t-tests, ANOVA, and OLS regression analyses were performed using Python (Python Software Foundation, Wilmington, DE, USA).

## Results

### Dental sealant utilization by demographic and institutional characteristics

The annual distribution of patients receiving dental sealants from 2016 to 2023 is presented according to sex, age group, region, and type of dental healthcare institution (Table 1). Inferential statistical tests were conducted to assess whether sealant utilization differed significantly across subgroups.

**Table 1:** Annual distribution of dental sealant utilization by demographic and institutional characteristics

Variables	2016	2017	2018	2019	2020	2021	2022	2023	Mean(%)	SD	*P*-value ^b^
Patient numbers (*N* = 5,853,477, unit: persons)											
Total	718,999	720,865	804,643	795,332	619,387	752,709	712,117	729,425	731,685(100)	57,436.53	
Sex											
Male	363,462	363,842	406,178	403,675	310,708	380,688	360,498	370,300	369,919(50.56)	29,865.46	0.058
Female	355,537	357,023	398,465	391,657	308,679	372,021	351,619	359,125	361,766(49.44)	27,606.72	
Age ^a^											
5–9 years	409,968	408,720	452,359	445,038	350,043	399,057	385,804	383,108	404,262(55.25)	33,345.45	< 0.001 ^c^
10–14 years	241,844	244,117	277,988	279,784	207,327	292,201	267,706	280,256	261,403(35.73)	28,188.45	
15–18 years	67,187	68,028	74,296	70,510	62,017	61,450	58,607	66,061	66,020(9.02)	5,153.47	
Region											
Metropolitan	387,427	387,668	429,380	434,397	315,842	417,433	391,120	398,888	395,269(54.02)	37,117.45	0.002
Rural	331,572	333,197	375,263	360,935	303,545	335,276	320,997	330,537	336,415(45.98)	22,355.06	
Type of dental healthcare institutions											
Clinic	687,956	689,190	769,690	760,651	591,945	721,797	681,838	698,690	700,220(95.70)	55,135.35	< 0.001
Hospital-level or Higher	31,043	31,675	34,953	34,681	27,442	30,912	30,279	30,735	31,465(4.30)	2,424.85	

^a^ Patient age was determined based on the exact age at the time of the visit, as recorded in the billing statement. Owing to this method, some patients may appear in multiple age groups if their birthdays are within the same year. For each year, the number of patients in each age group was estimated by applying the percentage within the 5–18-year age range to the total number of patients

^b^ Inferential statistics were used to assess whether observed differences across subgroups were statistically significant; statistical analyses were conducted using independent t-test and one-way ANOVA

^c^ Games–Howell post-hoc test was used for multiple comparisons because of the violation of the assumption of homogeneity of variance

Sealant utilization was similar between males and females, with males accounting for 50.56% and females for 49.44% of patients on average; this difference did not reach statistical significance (*P*=.058). In contrast, significant differences were observed across age groups (*P*<.001). Children aged 5–9 years (55.25%) constituted the largest proportion of sealant users, followed by those aged 10–14 years, whereas adolescents aged 15–18 years accounted for a substantially smaller proportion. Statistically significant regional differences were also identified (*P*=.002). Throughout the study period, metropolitan areas consistently had higher numbers of sealant users than rural regions. Regarding the type of dental healthcare institution, sealant utilization differed markedly across institution types (*P*<.001), with the vast majority of procedures performed in dental clinics (95.70%) rather than in hospital-level institutions.

### Online search trends for dental sealants

**Fig. 1:**
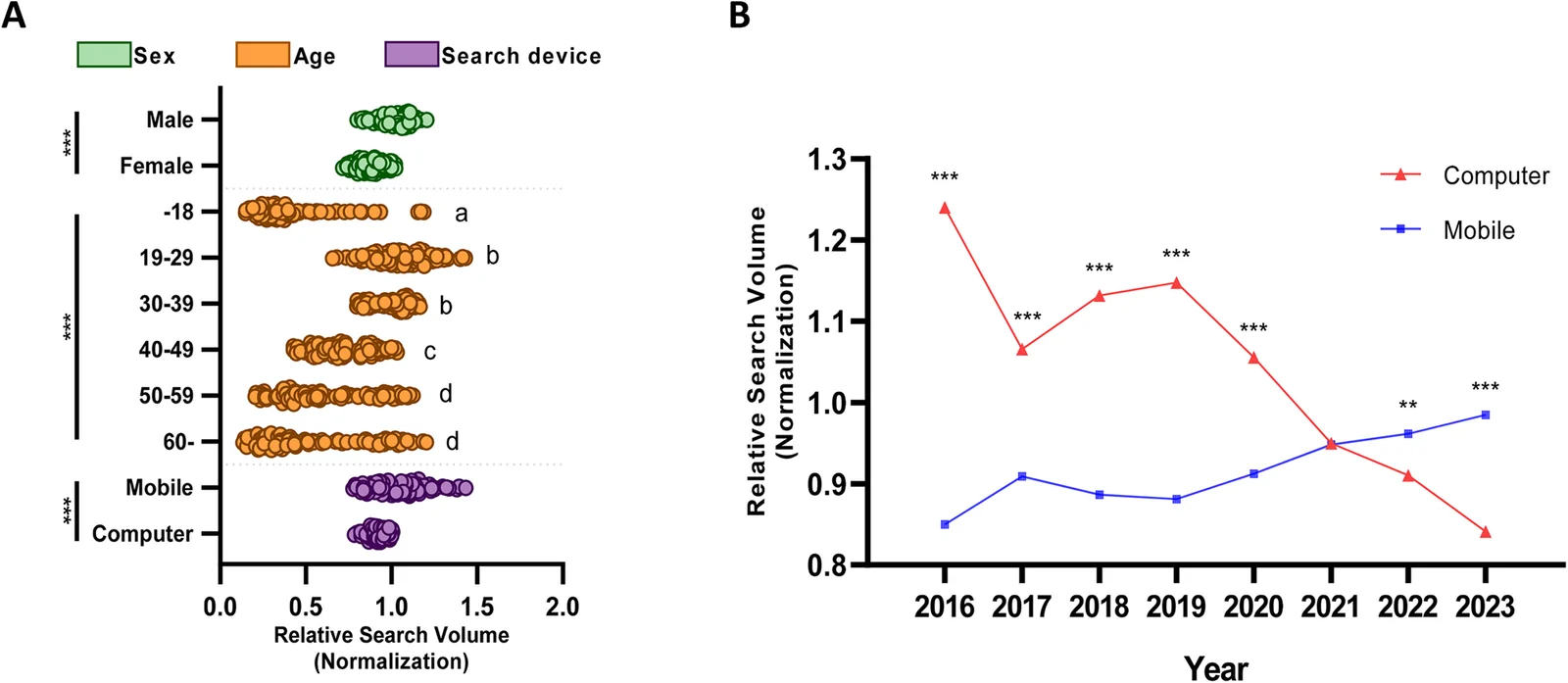
Online search trends for dental sealants. **A** Distribution of normalized relative search volume (RSV) by sex, age group, and search device. Each dot represents the normalized RSV for a single month. Different letters indicate statistically significant differences between age groups (*P*<.05). Group differences were assessed using *t* tests for sex and search device and one-way ANOVA for age groups; Games–Howell post hoc tests were applied due to unequal variances. Asterisks next to the y-axis factor labels indicate the overall significance of group differences for each factor (***P<.001). **B** Temporal trends in normalized RSV by search device from 2016 to 2023. Monthly RSV values (0–100) were obtained from Naver Data Lab (2016–2023) and normalized by dividing each monthly value by the total RSV across the study period. Asterisks indicate significant differences between computer- and mobile-based searches within each year (* *P*<.05, ** *P*<.01, *** *P*<.001)

RSV was analyzed to assess online search trends for dental sealants (Fig. 1A). Online search activity differed significantly by sex, age group, and search device, with statistically significant differences in normalized RSV observed across all examined subgroups. Normalized RSV differed significantly between males and females (*P*<.001), with a higher mean RSV observed among males. Age groups were defined according to the Naver Data Lab classification system. Significant differences were also identified across age groups (*P*<.001), and post hoc comparisons indicated distinct levels of online search activity across all age categories. Individuals aged 19–39 years demonstrated the highest RSV, whereas those younger than 18 years and those aged over 40 years exhibited lower search activity. In addition, normalized RSV varied significantly by search device (*P*<.001), reflecting differences in information-seeking behavior between computer- and mobile-based searches.

Figure 1B illustrates temporal trends in normalized RSV for dental sealants by search device from 2016 to 2023. In the earlier years of the study period, computer-based searches exhibited higher RSV than mobile-based searches, with statistically significant differences between devices from 2016 to 2020. During this period, computer-based RSV demonstrated a declining trend, whereas mobile-based RSV gradually increased over time.

From 2021 onward, the gap between search devices narrowed, and mobile-based searches surpassed computer-based searches in terms of RSV. Significant differences between devices were observed in the later years of the study period, with mobile searches demonstrating higher RSV than computer-based searches in 2022 (*P*<.01) and 2023 (*P*<.001). Overall, these findings indicate a shift in information-seeking behavior related to dental sealants from computer- to mobile-based platforms.

### Seasonal trends in dental sealant utilization and online search interest

**Fig. 2:**
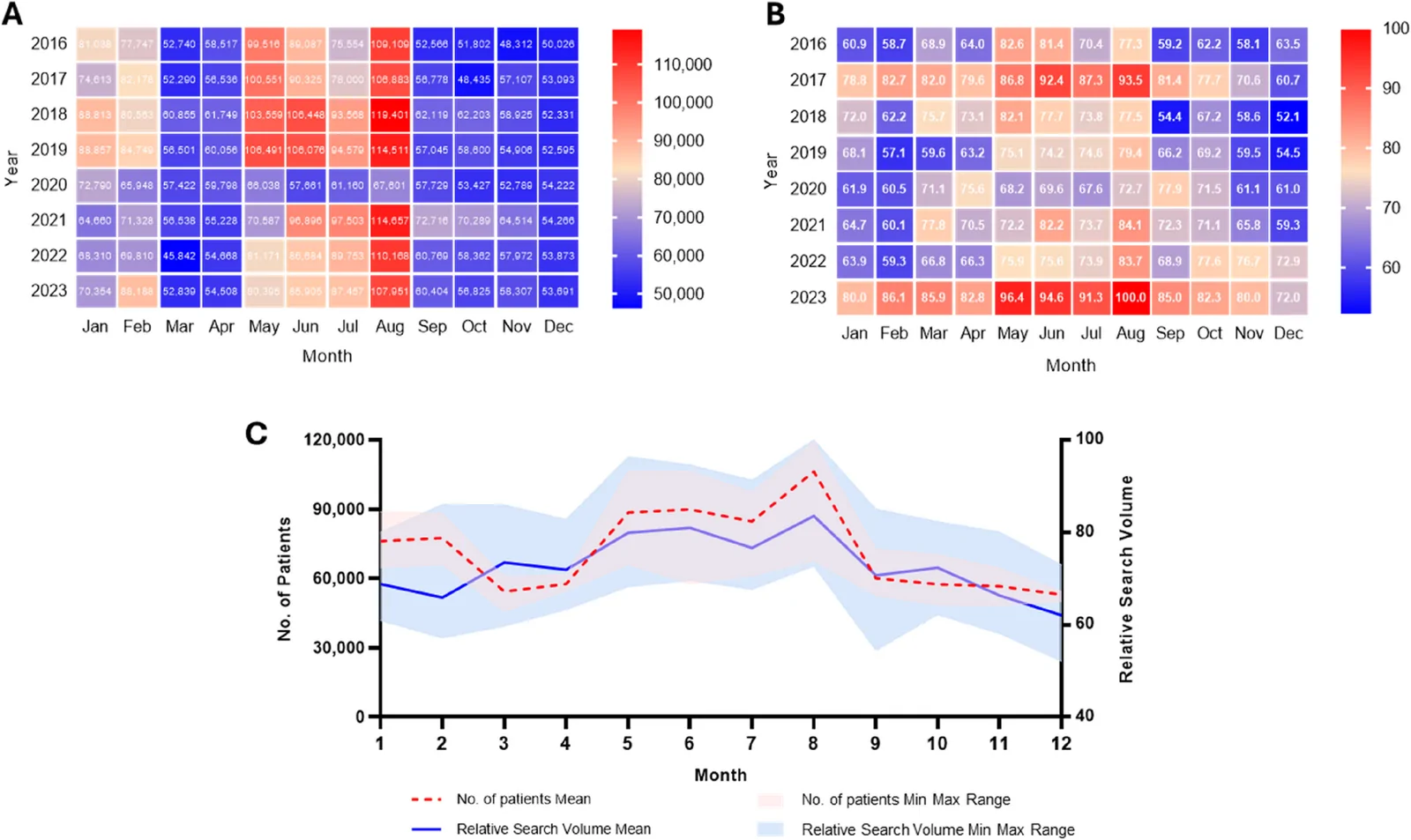
Longitudinal analysis of monthly patient numbers and relative search volume for dental sealants. **A** Number of patients who received dental sealant under the National Health Insurance from January 2016 to December 2023. **B** Relative search volume (RSV) for “Dental sealant” in Korean or English on the Korean portal NAVER from January 2016 to December 2023. RSV ranges from 0 to 100, representing the proportion of the highest observed search volume during the specified period. **C** Monthly trend of the mean number of patients (red dashed line) and the mean RSV for “dental sealant” (blue solid line) from January 2016 to December 2023. Shaded areas represent the minimum and maximum range for each month across the entire study period

Figure 2 presents longitudinal patterns of the monthly number of patients receiving dental sealants and RSV from 2016 to 2023. Sealant utilization consistently peaked between May and August, particularly during the summer vacation season (Fig. 2A). A pronounced decline in patient numbers was observed in 2020, coinciding with the COVID-19 pandemic, followed by a gradual recovery in subsequent years. Monthly RSV for “dental sealant” over the same period demonstrated a similar seasonal pattern. Online search interest, as measured by RSV, peaked in June (80.96) and August (83.53; Fig. 2B). Although RSV values fluctuated across years, recurrent peaks and troughs were consistently observed, indicating temporal variability in online search interest.

When both indicators were examined concurrently (Fig. 2C), the monthly mean number of patients and mean RSV demonstrated broadly concordant temporal trends. Periods with higher average RSV generally coincided with increased numbers of patients receiving dental sealants, whereas lower RSV levels corresponded to reduced utilization. Shaded ranges indicate substantial month-to-month variability across the study period. These parallel patterns suggest a temporal alignment between online search activity and sealant utilization, which was further evaluated using regression analysis.

### Descriptive statistics of variables used in the analysis

Table 2 presents the descriptive statistics for the variables used in the analysis of dental sealant utilization. The average monthly number of patients receiving sealants was approximately 71,857, with considerable regional variation (range: 45,842–119,401). RSV had a mean value of 72.69, indicating substantial variability in online interest related to dental sealants.

**Table 2:** Descriptive statistics of variables used in the regression analysis (N=1,632 ^a^)

Variable (Unit)	Mean	SD	Min	Max
Number of dental sealant patients (thousand persons)	71.86	19.32	45.84	119.40
Relative search volume (unitless)^b^	72.69	10.29	52	100
Population (thousand persons)^c^	420.41	449.85	44.32	2,038.17
Education level (thousand persons)^d^				
Low education	3,960.94	210.96	3,650	4,365
Medium education	10,288.00	160.86	9,916	10,515
High education	13,103.75	808.17	11,909	14,728
Annual household income (thousand KRW)	21,965.26	2,608.55	17,628	29,372
Number of dental clinics and hospitals (counts)	1,072.08	1,320.48	54	4,966
Oral examination season (dummy)^e^	0.43	0.49	0	1
Vacation season (dummy)^e^	0.33	0.47	0	1
Number of COVID-19 patients (thousand persons)	360.13	1,250.62	0	10,866.81

*SD *Standard deviation, *Min* Minimum, *Max* Maximum, *KRW* Korean won

^a^*N* = 1,632 represents region-month observations, calculated as 12 months × 8 years from 2016 to 2023 × 17 administrative regions of Korea, not individual patients

^b^ The relative search volumes (RSV) ranged from 0 to 100, representing the proportion of the highest observed search volume during the specified period

^c^ Population refers to the number of individuals aged 5–18 years at a regional level

^d^ Education refers to the number of employed individuals, classified according to their highest level of educational attainment at the regional level

^e^ Dummy variables for oral examinations and vacation seasons were coded as 1 = during the season and 0 = not during the season

The average population aged 5–18 years per region was 420,407, with some regions exceeding 2 million individuals. Educational attainment was categorized into three groups based on the OECD/UNESCO International Standard Classification of Education (ISCED 2011): low (ISCED 0–2), medium (ISCED 3–4), and high (ISCED 5–8) [31]. These categories correspond to less than middle school, high school, and college-level or higher education, respectively, and reflect regional differences in educational attainment. The average population sizes for these groups were 3,960.94, 10,288.00, and 13,103.75, respectively. Average annual household income was approximately 21.97 million Korean won (KRW), with substantial regional variation (mean (SD): 21,965 (2,609) thousand KRW; range: 17,628–29,372 thousand KRW). The number of dental clinics averaged approximately 1,072, ranging from 54 to nearly 5,000, reflecting differences in healthcare infrastructure.

School oral examination periods and vacation seasons were included as dummy variables in 43% and 33% of observations, respectively. The number of confirmed COVID-19 cases ranged from 0 to over 10.8 million, with a large SD, supporting its inclusion as a control variable to account for pandemic-related disruptions in healthcare utilization.

### Association between online search trends and dental sealant utilization

The effects of online search trends and related factors on sealant utilization were examined using regression analysis (Table 3). RSV, an indicator of online interest, was positively and significantly associated with sealant utilization (β = 26.92, *P*=.036), suggesting that increased online search activity was associated with higher numbers of patients receiving dental sealants. Specifically, holding other covariates constant, a 1-point increase in RSV (on a 0–100 scale) was associated with an estimated increase of approximately 26.9 sealant users in a given month.

**Table 3:** Multivariable ordinary least squares regression analysis of online search trends and dental sealant utilization

Variable	Coefficient (β)	Std. Error	t-value	*p*-value ^e^
Relative search volume ^a^	26.9231	12.821	2.100	0.036
Population ^b^	0.0077	0.003	2.992	0.003
Education level ^c^				
Low education	-23.0242	2.121	-10.854	< 0.001
Medium education	-0.0866	2.209	-0.039	0.969
High education	-3.0086	1.849	-1.627	0.104
Annual household income	0.2090	0.040	5.274	< 0.001
Number of dental clinics and hospitals	1.4972	0.830	1.804	0.071
Oral examination season ^d^	645.2878	294.304	2.193	0.028
Vacation season ^d^	1155.1078	278.933	4.141	< 0.001
Number of COVID-19 patients	-0.0002	0.000	-2.265	0.024

^a^ The relative search volumes (RSV) ranged from 0 to 100, representing the proportion of the highest observed search volume during the specified period

^b^ Population refers to the number of individuals aged 5–18 years at a regional level

^c^ Education refers to the number of employed individuals, classified according to their highest level of educational attainment at the regional level

^d^ Dummy variables for oral examinations and vacation seasons were coded as 1 = during the season and 0 = not during the season

^e^ The dependent variable was the number of patients insured under the National Health Insurance system who received dental sealants. R^2^ = 0.471, adj. R^2^ = 0.467; Durbin-Watson values = 2.382

Population size had a significant positive association with sealant utilization (*P*=.003). Relative to the reference category, medium and high educational attainment were not significantly associated with sealant utilization, whereas low educational attainment was significantly associated with lower utilization (*P*<.001). Household income demonstrated a positive association with sealant utilization (*P*<.001). Although the number of dental clinics was positively correlated with sealant utilization, this association did not reach statistical significance (*P*=.071).

Seasonal variables also significantly influenced sealant utilization. School oral examination periods (*P*=.028) and vacation seasons (*P*<.001) were significantly associated with increased sealant use. By contrast, the number of confirmed COVID-19 cases had a negative association with sealant utilization (*P*=.024). The regression model exhibited moderate explanatory power (R² = 0.471), and the Durbin–Watson statistic (2.382) suggested no substantial autocorrelation of residuals.

## Discussion

This study examined longitudinal trends in dental sealant utilization and online search activity in Korea from 2016 to 2023 by integrating clinical utilization data with population-level digital search indicators. The findings demonstrated that sealant utilization varied significantly by age, region, and socioeconomic characteristics and exhibited pronounced seasonal patterns. In parallel, online search interest in dental sealants, as measured by RSV, showed substantial heterogeneity across sex, age group, and search device, as well as marked temporal and seasonal variability. Importantly, periods of increased online search activity tended to coincide with higher sealant utilization, and multivariate regression analyses confirmed a significant positive association between RSV and sealant use, supporting the relevance of digital search trends as indicators of population-level interest in preventive dental services.

The number of individuals receiving dental sealants was lowest in 2020, likely reflecting the impact of the COVID-19 pandemic on dental service utilization. The highest utilization was observed in 2018, followed by 2019, indicating that sealant use was greatest during the pre-pandemic period. Although utilization subsequently recovered after 2020, it did not exceed the pre-pandemic peak. Children aged 5–9 years accounted for over 50% of all cases, highlighting the importance of sealant placement around the eruption of first permanent molars [32]. In contrast, individuals aged 15–18 years represented only a small proportion of cases, possibly because of reduced preventive focus at older ages or completion of permanent tooth eruption [33].

Sealant utilization was significantly higher in metropolitan areas than in rural regions. This regional pattern may be related to differences in dental service availability, accessibility, and parental awareness of preventive dental care rather than clinical need alone. In addition, 95.7% of sealant applications were performed in dental clinics, indicating that preventive dental services are delivered predominantly through primary dental care settings.

Analysis of RSV revealed significant differences according to sex, age, and search device. The higher RSV observed among males than among females suggests sex-based differences in information-seeking behavior. The highest search activity was observed among individuals aged 19–39 years, who are likely to include parents or guardians of school-aged children. This pattern is consistent with previous studies showing that younger and middle-aged adults more frequently use digital devices and seek health-related information online [34]. It also suggests that RSV may partly reflect parental information-seeking before preventive dental visits.

Regarding search device type, computer-based searches were initially more prevalent than mobile-based searches. However, mobile-based RSV steadily increased and eventually surpassed computer-based RSV by 2023. This shift likely reflects the widespread adoption of mobile devices and increasingly mobile-centered access to health information, particularly after the COVID-19 pandemic. These trends suggest the need for mobile-optimized, parent-oriented oral health information and sealant promotion strategies.

RSV, used as a nationwide monthly indicator rather than as regional data, reflects temporal changes in public interest. Seasonal patterns in RSV were closely aligned with increased clinical utilization, particularly during school oral examination periods and vacation seasons. Both RSV and sealant utilization peaked during the summer months, and RSV increased 1–2 months before sealant utilization peaked. This suggests that online search activity may reflect anticipatory information-seeking before parents or guardians schedule preventive dental visits.

Regression analysis confirmed a statistically significant positive association between RSV and sealant utilization. This finding supports the hypothesis that higher online interest is associated with increased uptake of preventive services, consistent with previous studies demonstrating associations between digital health information-seeking and health behaviors [10, 11, 35]. In the context of dental sealants, RSV may reflect caregivers’ awareness, perceived need, or intention to seek preventive care for children. However, online interest alone may not directly translate into service utilization; this relationship may be modified by socioeconomic resources, regional healthcare accessibility, and availability of dental institutions. This interpretation is supported by the positive associations of regional population size and average household income with sealant utilization and the negative association observed for lower educational attainment. Together, these findings suggest that digital search behavior can indicate population-level interest, but actual service uptake may depend on broader structural and socioeconomic conditions.

Sealant utilization increased during school oral examinations and vacation season. This temporal alignment suggests that online interest and actual service utilization may coincide, reflecting how digital search trends can serve as proxy indicators of seasonal demand. Together, these findings highlight the potential of RSV to predict public health service utilization and inform the strategic timing of preventive healthcare programs [36].

These findings have several policy implications. RSV may be used as a supplementary indicator to monitor population-level interest in preventive dental services and to identify periods when information-seeking or potential demand is likely to increase. Oral health promotion strategies could be timed to coincide with increased search activity, school oral examination periods, and vacation seasons. Regional differences in sealant utilization also suggest the need for targeted interventions in areas with lower service uptake, including improved access to primary dental care and parent-oriented education.

Several limitations should be considered. First, RSV is a relative measure and does not capture absolute search volume or individual-level interest. Second, major public health campaigns or promotional activities related to dental sealants were not included as separate variables because standardized monthly national-level data were not available across the entire study period. Third, RSV was analyzed as a nationwide monthly indicator rather than a region-specific measure; therefore, regional differences in online search behavior could not be directly compared with regional variations in sealant utilization. Finally, sealant utilization data were derived from NHI claims, which may not fully capture sealant applications provided as non-covered services or those delivered outside the claims system.

## Conclusions

In conclusion, dental sealant utilization in Korea was closely aligned with population-level online search activity and varied by demographic, socioeconomic, and seasonal factors, demonstrating the potential utility of online search trends as supplementary tools for public health planning and monitoring. RSV reflected temporal patterns of preventive service utilization, and the increasing predominance of mobile-based searches suggests a shift in how health information is accessed. Given the growing reliance on mobile devices and online platforms for health information seeking, these findings underscore the importance of providing accurate, evidence-based oral health information through digital channels. Strategic dissemination of reliable preventive dental information may not only enhance public awareness but also support informed decision-making and timely utilization of preventive dental services. Collectively, these findings support the use of mobile-centered digital indicators to inform the timing and design of preventive dental health policies and outreach strategies.

## Supplementary Information

Supplementary Material 1

## Data Availability

The datasets generated or analyzed during this study are available from the corresponding author upon reasonable request.

## Abbreviations

RSV: relative search volumes
