# Supplementary material for: Are online search trends associated with changes in dental sealant utilization?: focusing on key influencing factors
Source: BMC Oral Health. 2026 Jun 23;26:1524. doi: 10.1186/s12903-026-08733-5 (PMC13495230; doi:10.1186/s12903-026-08733-5)
Supplement: Supplementary file 1 [file 12903_2026_8733_MOESM1_ESM.docx]

Table S1. Definition and source of study variables

| Variables | Definition | Source | Ref. |
| --- | --- | --- | --- |
| Number of dental sealant patients | Number of patients who received sealants based on procedure code U2390. | HIRA | [7] |
| Relative Search Volume | Naver search volume (0–100) for keywords: “치면열구전색,” “치아홈메우기,” “실란트,” (in Korean), and "Sealant." | Naver Data Lab | [19] |
| Population | Number of individuals aged 5–18 per region | KOSIS | [24,25] |
| Number of dental institutions | Total number of dental clinics per region | KOSIS | [24,26] |
| Annual household income | Average income per household per region | KOSIS | [24,9,27] |
| Educational attainment | Proportion of working adults by education level | KOSIS | [24,9] |
| Vacation season (dummy) | Winter (Jan–Feb), Summer (Jul–Aug) | Defined in study | [28] |
| Oral examination season (dummy) | March–June (2016–17), April–July (2018–20), March–Sep (2021–23) | Defined in study | [29] |
| Number of COVID-19 cases | Monthly confirmed cases | NGO site | [30] |

These data were collected at the level of the 17 provinces and cities nationwide (Seoul, Busan, Daegu, Incheon, Gwangju, Daejeon, Ulsan, Sejong, Gyeonggi, Gangwon, Chungbuk, Chungnam, Jeonbuk, Jeonnam, Gyeongbuk, Gyeongnam, and Jeju).

HIRA, Health Insurance Review and Assessment; KOSIS, Korean Statistical Information Service; NGO, Nongovernmental organization.
